# Supplementary material for: Long non-coding RNAs as a source of new peptides
Source: eLife. 2014 Sep 16;3:e03523. doi: 10.7554/eLife.03523 (PMC4359382; doi:10.7554/eLife.03523)
Supplement: Supplementary file 1. — DOI: http://dx.doi.org/10.7554/eLife.03523.024 [file elife03523s001.docx]

**Supplementary File 1. Long non-coding RNAs as a source of new peptides. Ruiz-Orera et al., 2014.**

**A.** **Details on the number of coding transcripts associated with ribosomes.** codRNAe: Transcripts encoding experimentally validated proteins. codRNAne: Transcripts encoding non experimentally validated proteins. In the case of zebrafish codRNAe and codRNAne were grouped together due to the low number of experimentally validated proteins. Novel lncRNA: *de novo* assembled transcript that did not match or overlapped annotated genes in the corresponding species. In all cases, only transcripts expressed at > 0.5 FPKM or > 5 FPKM (yeast) were considered.

|  | **codRNAe** | **codRNAne** | **Pseudogene** | **Annotated lncRNA** | **Novel lncRNA** |
| --- | --- | --- | --- | --- | --- |
| **Mouse** | 5,462/5,465 (99.9%) | 8,734/8,780 (99.5%) | 1,124/1,158 (97.1%) | 218/253 (86.2%) | 172/223 (77.1%) |
| **Human** | 10,830/10,902 (99.3%) | 5,800/6,109 (94.9%) | 1,203/1,334 (90.2%) | 401/848 (47.3%) | 2/86 (2.3%) |
| **Zebrafish** | 11,643/12,595 (92.4%) | | 13/16 (81.3%) | 130/322 (40.4%) | 596/2,070 (28.8%) |
| **Fruit fly** | 1,450/1,450 (100.0%) | 6,581/6,595 (99.8%) | 11/11 (100.0%) | 15/16 (93.8%) | 7/12 (58.3%) |
| **Arabidopsis** | 3,874/3,888 (99.6%) | 15,005/15,274 (98.2%) | 67/73 (91.8%) | 55/61 (90.2%) | 38/78 (48.7%) |
| **Yeast** | 4,108/4,161 (98.7%) | 439/579 (75.8%) | 4/5 (80.0%) | 1/2 (50.0%) | 5/19 (26.3%) |

**B.** **ORF density and length in different types of transcripts.** ORFs were defined as starting with ATG, finishing with a STOP codon, and being at least 24 amino acids long. In the cases that two ORFs overlapped, the longest one was considered. Annotated coding transcripts (codRNA) correspond to mRNAs encoding experimentally validated proteins except for zebrafish, for which all transcripts annotated as coding were considered. Only transcripts with at least one ORF were considered. Annotated lncRNAs in yeast were not considered since only 2 of them were expressed in the sample. In all cases, only transcripts expressed at > 0.5 FPKM or > 5 FPKM (yeast) were considered. See Table 1 in main manuscript file for details on the experimental data. Av: average; Med: median; SD: standard deviation. ORF length is in nucleotides (nt).

| Open Reading Frame length | | | | | | | | | | | | | | | | | | |
| --- | --- | --- | --- | --- | --- | --- | --- | --- | --- | --- | --- | --- | --- | --- | --- | --- | --- | --- |
| **All ORFs** | **Novel lncRNA** | | | | | | **Annotated lncRNA** | | | | | | **codRNA** | | | | | |
|  | **ORFs/kb** | **ORF length (nt)** | | | | | **ORFs/kb** | **ORF length (nt)** | | | | | **ORFs/kb** | **ORF length (nt)** | | | | |
|  |  | **Av** | | | **Med** | **SD** |  | **Av** | | **Med** | | **SD** |  | **Av** | | **Med** | | **SD** |
| Mouse | 2.66 | | 173 | 130 | | 133 | 2.65 | 158 | 132 | | 173 | | 1.37 | | 516 | 148 | 949 | |
| Human | 2.65 | | 136 | 118 | | 59 | 2.69 | 166 | 127 | | 187 | | 1.36 | | 541 | 148 | 1059 | |
| Zebrafish | 2.64 | | 194 | 124 | | 346 | 2.59 | 204 | 124 | | 299 | | 1.33 | | 598 | 151 | 992 | |
| Fruit fly | 3.50 | | 139 | 114 | | 74 | 2.87 | 137 | 110 | | 56 | | 1.02 | | 992 | 240 | 1467 | |
| Arabidopsis | 3.80 | | 130 | 114 | | 46 | 2.64 | 136 | 114 | | 63 | | 0.95 | | 1060 | 870 | 1027 | |
| Yeast | 3.66 | | 136 | 126 | | 49 | - | - | - | | - | | 0.86 | | 1408 | 1185 | 969 | |

**C.** **Details on the number of non-coding transcripts associated with ribosomes.** The main lncRNA classes annotated in Ensembl gene annotations (v.70) are displayed in this table for mouse, human and zebrafish. In the rest of species all non-coding transcripts are annotated as ncRNA. In all cases only transcripts expressed at > 0.5 FPKM were considered.

|  | **lncRNA Annotated in Ensembl** | | | | | **lncRNA Novel** |
| --- | --- | --- | --- | --- | --- | --- |
| **Species dataset** | **lincRNA** | **Processed transcript** | **Non-coding retained intron** | **Antisense** | **Sense-intronic / overlapping** |  |
| **Mouse** | 130 / 152 | 60 / 67 | 9 / 9 | 19 / 25 | - | 172 / 223 |
| **Human** | 152 / 399 | 162 / 236 | 22 / 30 | 65 / 162 | 0 / 21 | 2 / 86 |
| **Zebrafish** | 17 / 54 | 108 / 256 | 4 / 8 | 1 / 4 | - | 596 / 2,070 |

**D. Homology hits for ORFs.** Primary ORFs from lncRNA_ribo and codRNAe, and longest ORFs from lncRNA_noribo with homologues in each of the species studied. Homology was inferred using BlastP and an E-value < 10^-4^ and searches were made against all predicted non-overlapping ORFs in lncRNAs and annotated CDS from protein-coding transcripts.

|  | **Primary ORF – lncRNA_ribo** | | | | | |
| --- | --- | --- | --- | --- | --- | --- |
|  | **Mouse** | **Human** | **Zebrafish** | **Fruit Fly** | **Arabidopsis** | **Yeast** |
| **Mouse** | 36 (9.1%) | 40 (10.2%) | 29 (7.4%) | 28 (7.1%) | 11 (2.8%) | 15 (3.8%) |
| **Human** | 32 (10.6%) | 50 (12.4%) | 31 (7.7%) | 26 (6.7%) | 17 (4.2%) | 13 (3.2%) |
| **Zebrafish** | 227 (31.3%) | 224 (30.9%) | 345 (47.5%) | 135 (18.6%) | 66 (9.0%) | 53 (7.3%) |
| **Fruit fly** | 0 (0.0%) | 0 (0.0%) | 1 (4.5%) | 0 (0.0%) | 0 (0.0%) | 0 (0.0%) |
| **Arabidopsis** | 0 (0.0%) | 0 (0.0%) | 0 (0.0%) | 0 (0.0%) | 6 (6.4%) | 0 (0.0%) |
| **Yeast** | 0 (0.0%) | 0 (0.0%) | 0 (0.0%) | 0 (0.0%) | 0 (0.0%) | 0 (0.0%) |

|  | **Longest ORF – lncRNA_noribo** | | | | | |
| --- | --- | --- | --- | --- | --- | --- |
|  | **Mouse** | **Human** | **Zebrafish** | **Fruit Fly** | **Arabidopsis** | **Yeast** |
| **Mouse** | 0 (0.0%) | 0 (0.0%) | 0 (0.0%) | 0 (0.0%) | 0 (0.0%) | 0 (0.0%) |
| **Human** | 7 (1.3%) | 49 (9.2%) | 3 (0.6%) | 1 (0.2%) | 0 (0.0%) | 4 (0.8%) |
| **Zebrafish** | 44 (6.1%) | 42 (2.6%) | 123 (7.5%) | 18 (1.1%) | 12 (0.7%) | 6 (0.4%) |
| **Fruit fly** | 0 (0.0%) | 0 (0.0%) | 0 (0.0%) | 0 (0.0%) | 0 (0.0%) | 0 (0.0%) |
| **Arabidopsis** | 0 (0.0%) | 0 (0.0%) | 0 (0.0%) | 0 (0.0%) | 1 (16.7%) | 0 (0.0%) |
| **Yeast** | 0 (0.0%) | 0 (0.0%) | 0 (0.0%) | 0 (0.0%) | 0 (0.0%) | 0 (0.0%) |

|  | **Primary ORF – codRNAe** | | | | | |
| --- | --- | --- | --- | --- | --- | --- |
|  | **Mouse** | **Human** | **Zebrafish** | **Fruit Fly** | **Arabidopsis** | **Yeast** |
| **Mouse** | - | 5392 (98.7%) | 5222 (95.6%) | 4613 (84.5%) | 3468 (63.5%) | 2883 (52.7%) |
| **Human** | 10638 (98.2%) | - | 10210 (94.3%) | 8829 (81.5%) | 6698 (61.8%) | 5484 (50.6%) |
| **Zebrafish** | 11115 (95.4%) | 10493 (90.1%) | - | 9256 (79.4%) | 6498 (55.8%) | 5242 (45.0%) |
| **Fruit fly** | 1350 (93.1%) | 1347 (92.9%) | 1296 (89.3%) | - | 999 (68.9%) | 862 (59.4%) |
| **Arabidopsis** | 2530 (65.3%) | 2520 (65.0%) | 2488 (64.2%) | 2403 (62.0%) | - | 2176 (56.2%) |
| **Yeast** | 2272 (61.5%) | 2265 (61.3%) | 2112 (57.2%) | 2138 (57.9%) | 2256 (61.1%) | - |

**E. GC content (%) in ORFs and complete sequences.** In transcripts associated with ribosomes the ORF corresponds to the primary ORF (see main manuscript file). We also defined different human gene deserts located in chromosomes chr7 and chr14 with different evolutionary properties (stable and flexible).

| **ORFs** | **Mouse** | **Human** | **Zebrafish** | **Fruit fly** | **Arabidopsis** | **Yeast** |
| --- | --- | --- | --- | --- | --- | --- |
| Flexible gene desert – chr4 | - | 37.52 | - | - | - | - |
| Stable gene desert – chr4 | - | 40.31 | - | - | - | - |
| Flexible gene desert – chr17 | - | 37.64 | - | - | - | - |
| Stable gene desert – chr17 | - | 40.01 | - | - | - | - |
| introns | 45.38 | 45.63 | 38.88 | 42.37 | 33.12 | 34.58 |
| lncRNA_noribo | 45.89 | 47.21 | 43.26 | 37.44 | 34.17 | 39.60 |
| lncRNA_ribo | 47.62 | 48.52 | 49.55 | 43.36 | 38.94 | 39.90 |
| pseudogene | 49.5 | 48.04 | - | - | - | - |
| codRNAne | 51.57 | 50.82 | - | 53.91 | 44.44 | 39.58 |
| codRNAe | 52.01 | 50.61 | 50.34 | 53.85 | 45.09 | 39.91 |

| **Sequences** | **Mouse** | **Human** | **Zebrafish** | **Fruit fly** | **Arabidopsis** | **Yeast** |
| --- | --- | --- | --- | --- | --- | --- |
| Flexible gene desert – chr4 | - | 34.54 | - | - | - | - |
| Stable gene desert – chr4 | - | 36 | - | - | - | - |
| Flexible gene desert – chr17 | - | 35.58 | - | - | - | - |
| Stable gene desert – chr17 | - | 38.49 | - | - | - | - |
| introns | 42.91 | 41.66 | 35.24 | 39.17 | 32.41 | 32.79 |
| lncRNA_noribo | 44.22 | 44.69 | 38.82 | 40.63 | 35.22 | 35.75 |
| lncRNA_ribo | 46.14 | 48.42 | 42.74 | 40.12 | 37.71 | 35.99 |
| pseudogene | 48.78 | 47.53 | - | - | - | - |
| codRNAne | 49.24 | 48.94 | - | 49.7 | 42.43 | 39.54 |
| codRNAe | 49.57 | 48.02 | 45.67 | 49.61 | 42.95 | 39.77 |

**F. PN and PS values for different sequence subsets.** PN: number of non-synonymous single nucleotide polymorphisms (SNPs). PS: number of synonymous SNPs. PN/PS: PN/PS ratio considering all sequences together. Youngest codRNA correspond to rodent-specific genes for mouse, primate-specific genes for human, *Actinopterygii*-specific (fish-specific) genes for zebrafish, See main manuscript file for more details.

| **PN** | **Mouse** | **Human** | **Zebrafish** |
| --- | --- | --- | --- |
| lncRNA_noribo | 362 | 812 | 94 |
| lncRNA_ribo | 1017 | 811 | 170 |
| codRNAne | 43064 | 59515 | 9131 |
| codRNAe | 23270 | 193293 |  |
| youngest codRNA | 254 | 159 | 709 |
|  |  |  |  |
| **PS** | **Mouse** | **Human** | **Zebrafish** |
| lncRNA_noribo | 142 | 271 | 57 |
| lncRNA_ribo | 511 | 305 | 209 |
| codRNAne | 87910 | 30059 | 18027 |
| codRNAe | 68969 | 107672 |  |
| youngest codRNA | 113 | 73 | 449 |
|  |  |  |  |
| **PN/PS** | **Mouse** | **Human** | **Zebrafish** |
| lncRNA_noribo | 2.55 | 2.66 | 1.65 |
| lncRNA_ribo | 1.99 | 3.00 | 0.81 |
| codRNAne | 0.49 | 1.98 | 0.51 |
| codRNAe | 0.34 | 1.80 |  |
| youngest codRNA | 2.25 | 2.18 | 1.58 |
|  |  |  |  |
| **95% confidence intervals PN/PS** | **Mouse** | **Human** | **Zebrafish** |
| lncRNA_noribo | 2.40-2.68 | 2.56-2.75 | 1.44-1.84 |
| lncRNA_ribo | 1.91-2.06 | 2.89-3.10 | 0.72-0.91 |
| codRNAne | 0.49-0.50 | 1.97-1.99 | 0.50-0.52 |
| codRNAe | 0.33-0.34 | 1.80-1.81 |  |
| youngest codRNA | 2.08-2.40 | 1.98-2.36 | 1.51-1.65 |
